# Ubiquitin-like processing of TUG proteins as a mechanism to regulate glucose uptake and energy metabolism in fat and muscle

**DOI:** 10.3389/fendo.2022.1019405

**Published:** 2022-09-29

**Authors:** Jonathan S. Bogan

**Affiliations:** ^1^ Section of Endocrinology and Metabolism, Department of Internal Medicine, Yale School of Medicine, New Haven, CT, United States; ^2^ Department of Cell Biology, Yale School of Medicine, New Haven, CT, United States; ^3^ Yale Center for Molecular and Systems Metabolism, Yale School of Medicine, New Haven, CT, United States

**Keywords:** insulin, glucose, GLUT4, membrane trafficking, ubiquitin-like, p97/VCP ATPases, adipocyte, muscle

## Abstract

In response to insulin stimulation, fat and muscle cells mobilize GLUT4 glucose transporters to the cell surface to enhance glucose uptake. Ubiquitin-like processing of TUG (Aspscr1, UBXD9) proteins is a central mechanism to regulate this process. Here, recent advances in this area are reviewed. The data support a model in which intact TUG traps insulin-responsive “GLUT4 storage vesicles” at the Golgi matrix by binding vesicle cargoes with its N-terminus and matrix proteins with its C-terminus. Insulin stimulation liberates these vesicles by triggering endoproteolytic cleavage of TUG, mediated by the Usp25m protease. Cleavage occurs in fat and muscle cells, but not in fibroblasts or other cell types. Proteolytic processing of intact TUG generates TUGUL, a ubiquitin-like protein modifier, as the N-terminal cleavage product. In adipocytes, TUGUL modifies a single protein, the KIF5B kinesin motor, which carries GLUT4 and other vesicle cargoes to the cell surface. In muscle, this or another motor may be modified. After cleavage of intact TUG, the TUG C-terminal product is extracted from the Golgi matrix by the p97 (VCP) ATPase. In both muscle and fat, this cleavage product enters the nucleus, binds PPARγ and PGC-1α, and regulates gene expression to promote fatty acid oxidation and thermogenesis. The stability of the TUG C-terminal product is regulated by an Ate1 arginyltransferase-dependent N-degron pathway, which may create a feedback mechanism to control oxidative metabolism. Although it is now clear that TUG processing coordinates glucose uptake with other aspects of physiology and metabolism, many questions remain about how this pathway is regulated and how it is altered in metabolic disease in humans.

## Introduction

Insulin stimulates glucose uptake from the circulation into fat and muscle cells. In individuals with insulin resistance, attenuation of this action contributes to metabolic diseases including type 2 diabetes. Therefore, substantial efforts have been directed to understand this hallmark insulin action. During fasting, when insulin concentrations are low, glucose uptake is restricted by the limited capacity for glucose transport across the plasma membrane. Under insulin-stimulated conditions, transport capacity is increased so that, in adipocytes, glucose uptake is increased by 10- to 20-fold. In muscle, the control of the overall rate of glucose uptake is distributed across multiple steps. These include glucose delivery by blood flow, transport into cells, and phosphorylation by hexokinase, which traps glucose intracellularly. In both cell types, the insulin-stimulated increase in glucose transport is pivotal to this regulation (1). Remarkably, the same molecular processes that increase glucose transport may also enhance glucose delivery (2). Yet, the physiologic regulation of these processes, and how they are affected in insulin resistance, is not well understood.

The primary mechanism by which insulin stimulates an increase in glucose transport capacity is the translocation of GLUT4 glucose transporters. GLUT4 is a 12-transmembrane domain -containing protein that is a member of a family of facilitative glucose transporters (3). It is expressed mainly in insulin-responsive cell types, such as fat and muscle. In the absence of insulin stimulation, GLUT4 is sequestered intracellularly, so that it is largely excluded from the plasma membrane and glucose uptake is restricted. Upon insulin addition, fat and muscle cells mobilize GLUT4 to the plasma membrane. This response occurs within minutes and can increase plasma membrane GLUT4 by as much as ~20-fold in primary adipocytes. In muscle, the response is less, and GLUT4 in the sarcolemma and T-tubules is increased by 2- to 4-fold (4). The process by which GLUT4 is acutely redistributed to plasma membranes, termed “GLUT4 translocation,” is widely considered to be both cell type -specific and GLUT isoform -specific.

The intracellular GLUT4-containing vesicles that are mobilized to the plasma membrane upon insulin stimulation have been called “GLUT4 Storage Vesicles” (GSVs) or “Insulin-Responsive Vesicles” (IRVs) (1, 5). These vesicles are not well understood. GSVs have been defined primarily in negative terms: they are distinct from endosomes, lysosomes, Golgi cisternae, and other organelles, and they are selectively mobilized to the cell surface by acute insulin stimulation. Although GSVs accumulate in unstimulated fat and muscle cells, it is debated whether these vesicles are present, at least to some degree, in “undifferentiated” fibroblasts and other cell types (6). Part of this debate relates to the nomenclature of what constitutes a “GSV.” Despite this uncertainty, understanding these vesicles is critical, because GSV mobilization is main action by which insulin stimulates glucose uptake.

## GLUT4 storage vesicles are regulated by TUG proteins

**Figure 1:**
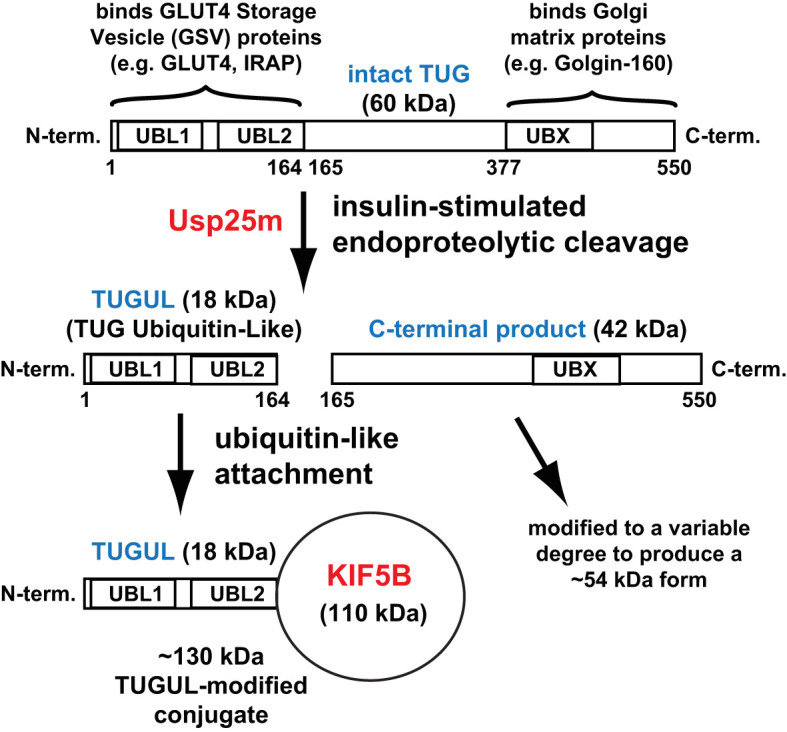
Ubiquitin-like proteolytic processing of TUG. Intact TUG protein is 60 kDa and has the domain structure that is shown. UBL1 and UBL2 are ubiquitin-like domains and UBX is a ubiquitin-regulatory X domain. The residues in the protein are numbered, as indicated, according to the mouse sequence. The N-terminal region binds GLUT4 Storage Vesicle (GSV) proteins, and the C-terminal region binds Golgi matrix proteins, as indicated. In fat and muscle cells, insulin stimulates endoproteolytic cleavage of intact TUG at the bond linking residues 164 and 165. Cleavage is mediated by the Usp25m protease and produces TUGUL (TUG Ubiquitin-Like), an 18 kDa ubiquitin-like protein modifier. The C-terminal product is 42 kDa and it is modified to a 54 kDa form to a variable degree. This modification is not understood. In 3T3-L1 adipocytes, TUGUL is covalently attached to a single target protein, the 110 kDa KIF5B kinesin motor, to produce a 130 kDa conjugate. In muscle, the TUGUL-modified (“tugulated”) protein has not been identified, and it is hypothesized that it is KIF5B or another kinesin motor. TUG cleavage liberates the GLUT4 storage vesicles from the Golgi matrix, and tugulation of the kinesin causes the movement of these vesicles toward the cell surface on these microtubule motors. The figure is modified from Habtemichael EN, et al., (11).

Here, we define GSVs as GLUT4-containing vesicles that are trapped intracellularly by TUG proteins, and that are mobilized by insulin-stimulated TUG endoproteolytic cleavage. TUG (Tether, containing a UBX domain, for GLUT4) was identified in a genetic screen for regulators of GLUT4 trafficking (7). It is also called ASPSCR1 or ASPL, reflecting its involvement in chromosomal translocations in alveolar soft part sarcomas, and UBXD9 or UBXN9, reflecting its membership in a family of UBX domain -containing proteins (8, 9). In fat and muscle cells, intact TUG undergoes ubiquitin-like proteolytic processing (10–13). Processing is stimulated by insulin and generates an N-terminal product, TUGUL (TUG Ubiquitin-Like), that is a ubiquitin-like protein modifier, as shown in **Figure 1**. Several lines of evidence now support the idea that TUG proteolytic processing is the main biochemical mechanism by which insulin regulates GSVs:

### A kinetically-defined pool of insulin-responsive GLUT4 is regulated by TUG

**Figure 2:**
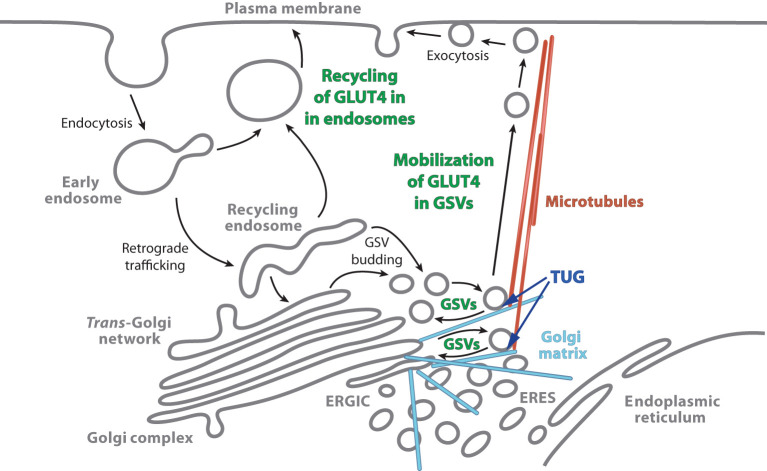
Actions by which insulin stimulation causes increased plasma membrane GLUT4. Insulin stimulates the mobilization of GLUT4 Storage Vesicles (GSVs), and also redirects endocytosed GLUT4 so that it returns directly to the plasma membrane from endosomes. In unstimulated cells, most GLUT4 accumulates in GSVs. These vesicles are trapped at the Golgi matrix by the action of intact TUG with other proteins, and they may participate in a cycle involving internal membranes (as described in the text). Insulin stimulates the acute proteolytic processing of TUG to mobilize the GSVs on microtubule motors directed toward the cell surface. The GSVs fuse at the plasma membrane, and the liberated GLUT4 is then able to populate the endosomal system. During ongoing insulin exposure, recycling of GLUT4 in endosomes becomes a significant source of GLUT4 arriving at the plasma membrane. The model that is depicted is based primarily on studies in adipocytes; data suggest that it holds in muscle as well. In skeletal muscle, data from TUG knockout mice imply that other sites at which insulin modulates GLUT4 trafficking are less important for its overall distribution at the cell surface. ERGIC, Endoplasmic Reticulum–Golgi Intermediate Compartment; ERES, Endoplasmic Reticulum Exit Sites. The figure is modified from Bogan JS, (1).

Early data, using mathematical models of GLUT4 trafficking, supported the hypothesis that GSVs contain the GLUT4 that is translocated to the plasma membrane within the first 5-8 min. after acute insulin addition to 3T3-L1 adipocytes (14, 15). During ongoing insulin exposure, >15-20 min. after acute insulin addition, the pool of GSVs is depleted and modeling suggested that GLUT4 may arrive at the cell surface from endosomes. As described below, this idea has now been supported experimentally (16, 17). These two pathways by which insulin recruits GLUT4 to the plasma membrane are illustrated in **Figure 2**. The size of the initial burst of GLUT4, translocated to the cell surface after acute insulin stimulation, corresponds to the size of the sequestered pool of GSVs, and was increased by TUG overexpression and decreased by expression of a truncated TUG fragment (7).

### TUG accounts for GLUT isoform specificity

TUG binds specifically to GLUT4 and not GLUT1, and insulin stimulates the dissociation of intact TUG from GLUT4 in fat and muscle cells (7, 18). The number of TUG-GLUT4 complexes that dissociate determines the size of the initial burst of GLUT4 that is translocated to the plasma membrane after acute insulin stimulation (7). Dissociation of intact TUG from GLUT4 results from TUG endoproteolytic cleavage (10, 11). Proteins that are not enriched in GSVs, such as GLUT1 and transferrin receptor (TfR), are not regulated by TUG (7, 19). Binding of GLUT4 to TUG is direct and is mediated by GLUT4 domains that confer insulin-responsive trafficking (19, 20). TUG also binds and regulates the insulin-responsive aminopeptidase, IRAP, a single transmembrane domain protein that cotraffics with GLUT4 in GSVs (21, 22).

### TUG regulates nonendosomal GLUT4

By confocal microscopy of 3T3-L1 adipocytes, TUG localizes on intracellular, punctate vesicles that stain for GLUT4, but not TfR (7). Consistent with the idea that these nonendosomal, GLUT-containing vesicles are GSVs, fewer of these punctae were apparent in insulin -treated cells, compared to unstimulated cells. More strikingly, manipulation of TUG altered the fraction of intracellular GLUT4 in nonendosomal vesicles (19). TUG overexpression caused an expansion of the pool of GLUT4 not colocalized with TfR, as observed by confocal microscopy. Conversely, expression of a truncated fragment of TUG caused both insulin -independent GLUT4 translocation to the cell surface and complete overlap of the remaining intracellular GLUT4 with TfR, an endosomal marker. These data further support the idea that TUG binds and regulates GLUT4 that is present in GSVs and not endosomes (**Figure 2**).

### TUG disruption mimics effects of insulin on GLUT4 targeting and stability in 3T3-L1 adipocytes

Depletion of TUG, or expression of a truncated fragment, caused effects on GLUT4 translocation and glucose uptake that were similar in magnitude to those of insulin stimulation (19). There were minimal further effects of insulin in cells with disruption of TUG, arguing that TUG acts at the main site at which insulin regulates GLUT4 and glucose uptake, that is, the pool of GSVs. Similar results were obtained for the rate of vesicle exocytosis, measured using total internal reflection fluorescence microscopy in living cells containing fluorescent GSV cargoes (16). In addition, TUG disruption mimicked effects of insulin to reduce GLUT4 protein half-life by accelerating its degradation in lysosomes (19). The data support the idea that TUG-mediated sequestration of GLUT4 in GSVs prolongs its half-life, and further imply that GSVs sequester GLUT4 away from lysosomes as well as from the plasma membrane.

### TUG regulates vesicles with the size and cell type -specificity of GSVs

Total internal reflection fluorescence microscopy of living 3T3-L1 adipocytes showed that TUG regulates the exocytic translocation of vesicles that are the same size as GSVs, which are ~60 nm in diameter (16). These vesicles were clearly distinct from endosome-derived vesicles, which were ~150 nm in diameter. The ~60 nm diameter vesicles are cell type -specific, as they were observed in differentiated 3T3-L1 adipocytes but not in undifferentiated 3T3-L1 cells. The data are consistent with earlier electron microscopy studies showing that GSVs have a characteristic diameter of 50–70 nm, and that the biogenesis of these vesicles is cell type -specific and dependent the upon expression of a sorting receptor, sortilin (5, 21, 23, 24). The results further show that GSVs are translocated to the plasma membrane after acute (3-6 min.) insulin treatment, but that GLUT4 recycles to the plasma membrane from endosomes during ongoing (>15 min.) insulin exposure (16). Subsequent work further supports this idea and shows that, in adipocytes, GLUT4 in GSVs translocates to the plasma membrane together with Rab10, whereas GLUT4 in endosomes translocates together with Rab14 (17, 25). These results fit well with the kinetics described above and with the model shown in **Figure 2**.

### TUG regulates GSV-specific cargoes in addition to GLUT4

In adipocytes, GSVs contain a distinct set of proteins, including IRAP, LRP1, sortilin, VAMP2, and likely VAMP3, syntaxin-6 (Stx6) and TRARG1 (a.k.a. Tusc5) (21, 26–31). In addition to GLUT4, TUG-bound vesicles contain IRAP, LRP1, VAMP2, VAMP3, and Stx6, and these cargoes are mobilized by insulin-stimulated TUG cleavage in 3T3-L1 adipocytes (11, 13, 22). As well, coexpression of sortilin enhances Usp25m-mediated TUG cleavage, as described further below (11). Conversely, TUG does not appreciably regulate the localization of several non-GSV proteins, including GLUT1, TfR, insulin receptor β-chain, various SNAREs, the α1 subunit of Na^+^-K^+^ ATPase, and Caveolin (7, 10, 12, 13, 16, 19, 22).

### TUG cleavage is a plausible mechanism to regulate GSVs

The N-terminal region of TUG binds to GSV proteins, GLUT4 and IRAP, and the C-terminal region binds to Golgi matrix proteins including Golgin-160, PIST (a.k.a. GOPC), and ACBD3 (a.k.a. GCP60) (10, 19, 32). Intact TUG traps the vesicles at the Golgi matrix, possibly by constraining a cycle of membrane budding and fusion (**Figure 2**). Insulin-stimulated TUG cleavage separates vesicle- and matrix- binding regions of TUG, as shown in **Figure 1**, and is required for GLUT4 and IRAP translocation (10, 11). The number of TUG molecules that are cleaved is approximately equal to the number of GSVs that are translocated (10). Furthermore, data imply that the N-terminal cleavage product, TUGUL, is covalently attached to a kinesin motor, KIF5B, that carries GLUT4 to the cell periphery in adipocytes (11, 33). TUG cleavage is required to load GLUT4 on to these motors (11). The TUGUL-modified protein in muscle has not been identified but seems likely to be KIF5B or another kinesin motor. The data imply that TUG processing not only liberates the vesicles, but also activates their microtubule-based movement to the cell surface (**Figure 2**).

### Other GSV-regulating proteins are present on TUG-bound vesicles

**Figure 3:**
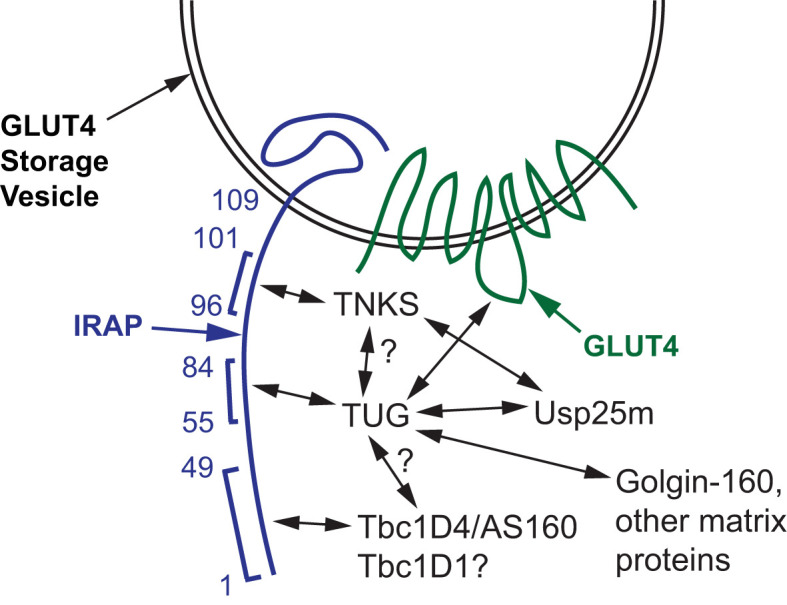
Proteins that bind and regulate GLUT4 Storage Vesicles (GSVs). A GSV membrane containing IRAP and GLUT4 is shown, and amino acid residues in the cytosolic N-terminus of IRAP are numbered. Tankyrase (TNKS), TUG, and Tbc1D4 (AS160) bind to adjacent peptides in IRAP, as indicated by brackets. TUG also binds the large intracellular loop of GLUT4, and both TUG and TNKS bind Usp25m. Direct binding of TUG to TNKS and to Tbc1D4 has not been shown; these hypothesized interactions are indicated with question marks. Tbc1D1 binds the cytosolic domain of IRAP (residues 1-109), but this interaction has not been localized further; the question mark indicates that it is hypothesized to bind IRAP residues 1-49 similar to Tbc1D4. TUG binds to Golgin-160 and other Golgi matrix proteins through its C-terminus, which is required to trap the vesicles intracellularly in the absence of insulin stimulation.

In addition to the GSV cargoes noted above, cytosolic proteins are recruited to GSVs and regulate these vesicles. These include the Rab GTPase Activating Proteins (GAPs) Tbc1D4 (a.k.a. AS160) and Tbc1D1, the poly(ADP-ribose) polymerase tankyrase (TNKS1 and TNKS2), and the protease Usp25m (11, 26, 34–39). In 3T3-L1 adipocytes, both Tbc1D4 and Usp25m are present on TUG-bound vesicles in unstimulated cells (11). Tankyrase (TNKS), Tbc1D4 and TUG bind to adjacent sites within the cytosolic portion of IRAP, as shown in **Figure 3**, and these proteins may also interact directly (22, 40, 41). TNKS recruits Usp25m, which regulates GLUT4 trafficking and is required for insulin-stimulated TUG endoproteolytic cleavage, as described further below (40, 42–44). In transfected cells, Tbc1D4 enhances the effect of Usp25m to cause TUG cleavage, further supporting the idea that these proteins act together to mobilize GSVs (11).

### TUG deletion mimics insulin effects on GLUT4 targeting and glucose uptake in skeletal muscle

In muscle-specific TUG knockout (MTKO) mice, a conditional allele was used to delete TUG in muscle (13). In the fasting mice, TUG deletion caused a 3.6-fold increase in GLUT4 abundance in T-tubule -enriched membrane fractions prepared from quadriceps muscles. This effect was not significantly different from that of insulin, which caused a 4.1-fold increase in GLUT4 in T-tubules of wildtype controls. More important, there was no effect of insulin to stimulate any additional GLUT4 translocation in MTKO mice; in both fasted and insulin-stimulated muscles, T-tubule GLUT4 was increased by 3.6-fold compared to fasted controls. Muscle-specific glucose uptake was increased by 2.0-fold in gastrocnemius and 1.8-fold in quadriceps muscles of fasting MTKO mice, compared to controls, as assessed *in vivo* using an infused tracer (13). These effects resulted in reduced fasting glucose concentrations, reduced fasting insulin concentrations, and increased whole-body fasting glucose turnover. Of note, insulin stimulated an ~80% reduction in the abundance of intact TUG in homogenates of quadriceps muscles of wildtype mice; experiments using muscle lysates revealed a smaller decrease in intact TUG abundance, together with generation of the C-terminal cleavage product (13). The data support the idea that TUG regulates the main site at which insulin acts to stimulate GLUT4 translocation in skeletal muscle, and that this can account for a substantial fraction of insulin’s effect to stimulate glucose uptake *in vivo*.

As an aside, the predicted itinerary of GLUT4 is not identical in muscles of fasting MTKO mice and in insulin-stimulated wildtype mice. In MTKO mice, much of the endocytosed GLUT4 will follow the retrograde trafficking pathway depicted in **Figure 2**, since phosphatidylinositol-3-kinase (PI3K) and Akt are not activated, as discussed in the next section. It is anticipated that this GLUT4 will enter budding GSVs, but that these vesicles will not be trapped intracellularly by TUG and Golgi matrix proteins, and that they instead fuse with the plasma membrane (16, 17, 45). This is distinct from the effect of insulin stimulation, which causes the recycling of GLUT4 from endosomes, in addition to the release of GSVs, as implied by data from adipocytes. It remains the case that insulin caused no further effect in muscles with TUG deletion, implying that the release of GSVs is the main site of action in muscle *in vivo*.

The results inform the debate about whether GLUT4 is regulated by a “static retention” or a “dynamic equilibrium” mechanism (1, 4). In static retention model, there is one main site of insulin action, the GSVs, which accounts for the bulk of its overall effect to mobilize GLUT4 to the plasma membrane. The dynamic equilibrium model posits that the overall change in GLUT4 distribution results from the cumulative effect of insulin to modulate trafficking at multiple sites. It has been argued that replating of 3T3-L1 adipocytes disrupts the intracellular sequestration of GSVs, so that the dynamic model reflects specific experimental manipulations (46). Supporting this view, loss of cell adhesions causes dramatic alteration of trafficking at the Golgi, which likely impacts TUG-regulated pathways (47). Ultimately, what matters is how these pathways are regulated *in vivo*. In muscles with disruption of a specific TUG-regulated step in GLUT4 trafficking, insulin had no further effect to redistribute GLUT4 (13). The data support the static retention model, at least in muscle. Other data also support the static retention model in adipocytes (11, 19, 48). This does not imply that the GSVs themselves are entirely static, and they may participate in a cycle of budding and fusion involving internal membranes (**Figure 2**). Yet, the implication is that effects of insulin at other sites (e.g. to reduce GLUT4 endocytosis, to promote GSV budding, or to accelerate GLUT4 vesicle exocytosis at the plasma membrane) contribute only minimally to the physiologic regulation of GLUT4 distribution.

### The TUG proteolytic pathway coordinates glucose uptake with other aspects of physiology and metabolism

**Figure 4:**
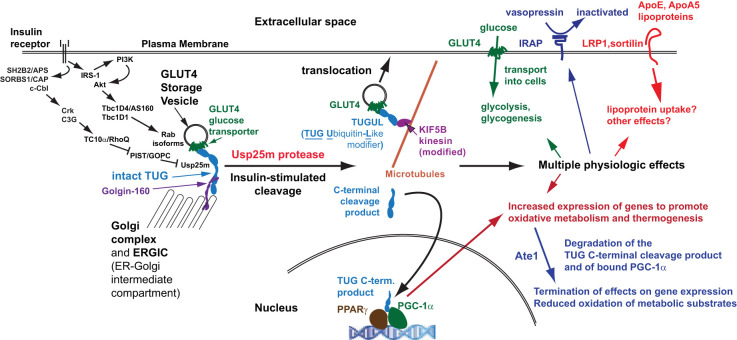
Coordinated regulation of distinct physiologic effects. Insulin signals through at least two signaling cascades to mobilize GLUT4 storage vesicles (GSVs). One involves Akt and the RabGAPs, Tbc1D4 and Tbc1D1, which regulate distinct Rab proteins on the GSVs (and on other membranes, such as endosomes). A second pathway involves the Rho family GTPase TC10α, which is coupled through its effector PIST to the TUG protease, Usp25m. Insulin stimulates TUG endoproteolytic cleavage to liberate the GSVs from Golgi matrix proteins, including Golgin-160 and others not shown. The N-terminal cleavage product, TUGUL, is a ubiquitin-like modifier. In adipocytes, TUGUL is attached to KIF5B, a microtubule motor that carries the GSVs to the cell surface; in muscle, this or another kinesin may be involved. Fusion of these vesicles at the cell surface results in the insertion of their specific cargo proteins into the plasma membrane. These include GLUT4, which promotes glucose uptake, IRAP which cleaves and inactivates circulating vasopressin, and LRP1 and sortilin, which bind the lipoproteins ApoE and ApoA5 and may regulate lipid metabolism. These proteins may have other effects as well. The TUG C-terminal product is extracted from the Golgi matrix by the action of p97 ATPases and is targeted to the nucleus. It binds the transcriptional regulator PPARγ and its co-factor PGC-1α, and stimulates the expression of genes to promote lipid oxidation and thermogenesis. The stability of the C-terminal product is regulated by the Ate1 arginyltransferase, and the product confers Ate1-dependent stability on PGC-1α. Degradation of these proteins makes their thermogenic effect transient, consistent with the transient increase in thermogenesis that is observed after meals.

In muscle, TUG cleavage coordinates distinct physiologic effects due to translocation of GSV cargo proteins, and due to actions of the TUG C-terminal cleavage product, as depicted in **Figure 4** and in **Table 1**, and as discussed further below. In addition to GLUT4, IRAP is translocated to T-tubules, as noted above. This causes accelerated degradation of circulating vasopressin, an IRAP substrate, and may enhance blood flow in muscles that have increased metabolic activity (13, 22). Possibly, altered targeting of IRAP contributes to hypertension in insulin-resistant individuals (2). Other GSV cargoes, such as LRP1 and sortilin, bind to ApoE and ApoA5, and we speculate that targeting of these proteins regulates lipid metabolism (2, 27, 49). Finally, the TUG C-terminal product controls oxidative metabolism and energy expenditure, as discussed below (13). These observations further support the idea that TUG-regulates GSVs, and that this is a central site for insulin action in fat and muscle.

**Table 1:** Physiologic effects of TUG ubiquitin-like proteolytic processing.

Molecular action	Physiologic effect	Role in pathophysiology
**Effects of vesicle translocation**		
GLUT4 insertion at the plasma membrane	glucose uptake	insulin resistance in muscle and adipose
IRAP insertion at the plasma membrane	vasopressin inactivation	contribution to hypertension (?)
LRP1, sortilin insertion at the plasma membrane	role in lipid metabolism (?)	contribution to dyslipidemia (?)
**Effects of the TUG C-terminal cleavage product**		
Expression of genes that mediate thermogenesis (e.g. Sln, Ucp1)	increased energy expenditure	contribution to obesity (?) contribution to diabetes risk, with PPARγ2 Pro12Ala (?)

Effects on glucose uptake, vasopressin inactivation, and energy expenditure have been demonstrated in mouse models. Other physiologic and pathophysiologic effects are hypothesized and are so indicated using question marks. See text for details.

## Insulin signaling upstream of GLUT4

Insulin signals through several pathways upstream of GLUT4, which likely regulate different steps in GLUT4 trafficking and which are detailed in recent reviews (4, 15, 50, 51). The pathway that has been the focus of most study proceeds by a phosphorylation cascade from the insulin receptor to insulin receptor substrate (IRS) proteins, phosphatidylinositol-3-kinase (PI3K), and Akt proteins (**Figure 4**). Activated Akt phosphorylates the RabGAPs, Tbc1D4 and Tbc1D1, which causes the dissociation of these proteins from IRAP (39, 52, 53). This likely occurs on both GSVs and endosomes, and removal of the GAP activity from these vesicles is thought to activate specific Rab GTPases to modulate vesicle traffic. These Rab GTPases include Rab10 in adipocytes and Rab8A and Rab13 in myocytes, which are present on GSVs, and Rab14, which is present on endosomes in both fat and muscle cells (17, 26, 39). Rab10 may also reside on membranes from which GSVs are formed, as it acts at least in part through Sec16A to promote GSV budding (54). Rab8A acts through MyoVa and is thought to be involved in transferring GSVs from microbubules to actin filaments, enabling the subsequent action of Rab13 through MICAL-L2 and α-actinin-4 on cortical actin (4, 55, 56). Other downstream effectors of these Rab proteins are not well defined.

The insulin signaling pathway that triggers TUG cleavage is PI3K-independent and requires the Rho family GTPase TC10α, also called RhoQ (10, 11). This pathway is also independent of IRS proteins. Activated insulin receptors bind SH2B2 (a.k.a. APS), which recruits SORBS1 (CAP) and c-Cbl (57–59). Cbl is then phosphorylated by the insulin receptor and recruits the adaptor Crk and the GTP Exchange Factor (GEF) C3G (RAPGEF1), which activates TC10α (51, 60). Compartmentalization of TC10α on lipid rafts, which may be present on internal membranes, is required for its ability to act in GLUT4 translocation (61). Knockdown experiments show that TC10α is required for insulin-stimulated GLUT4 translocation and glucose uptake, as well as for TUG cleavage, in 3T3-L1 adipocytes (10, 62). Activated TC10α signals through effectors, including the exocyst complex subunit Exo70, to stimulate GLUT4 fusion with the plasma membrane (63). The relevant TC10α effector for TUG cleavage is PIST (PDZ domain protein that interacts specifically with TC10, a.k.a. GOPC), which binds TUG directly (10, 64). Of note, PIST also binds syntaxin-6, which is present in TUG-bound vesicles, and Golgin-160, which helps to anchor these vesicles at the Golgi matrix (10, 32, 65, 66).

Data imply that PIST is a negative regulator of TUG cleavage in unstimulated cells, and that binding of activated TC10α relieves this inhibition (**Figure 4**). A truncated, artificial C-terminal fragment of TUG, “UBX-Cter” (residues 377-550), causes GLUT4 translocation and glucose uptake in unstimulated 3T3-L1 adipocytes, similar to TUG RNAi (19). Initial studies to disrupt TUG action in skeletal muscle used transgenic mice expressing this fragment (12). These “UBX mice” (formerly, mTUG^UBX-Cter^ mice) also have GLUT4 translocation and increased muscle-specific and whole-body glucose uptake during fasting. Indeed, quadriceps-specific glucose uptake was increased 2.7-fold during fasting in these mice, and there was no further effect of insulin to stimulate uptake; glycogen stores were also increased by 1.7- to 2.1- fold in various muscles (12). Mechanistically, UBX-Cter binds Golgin-160, ACBD3, and PIST, and it was initially thought to compete with intact TUG for binding to a GSV “anchoring site.” Yet, UBX-Cter protein was present at low abundance compared to intact TUG, implying a noncompetitive mechanism. A possibility was that UBX-Cter causes proteasomal degradation of a binding protein, together with itself. Consistent with this idea, PIST protein abundance was markedly depleted by expression of UBX-Cter. Simultaneously, intact TUG was cleaved constitutively, and was no longer subject to insulin regulation. The data imply that PIST normally acts as a brake on TUG cleavage; its removal from a TUG-GLUT4 complex, or its modulation by binding of GTP-TC10α, relieves this inhibitory effect.

The idea that binding of activated TC10α to PIST removes a brake on the TUG protease, Usp25m (see below), must remain speculative at present. Studies in which PIST is knocked down or deleted have not been performed and, although binding of PIST and TUG is direct, biochemical effects of PIST on Usp25m have not been studied. It remains possible that PIST and UBX-Cter are rendered insoluble rather than degraded. Either way, TUG cleavage and GLUT4 translocation result. Of note, the PIST isoform identified in skeletal muscle corresponds to nPIST, which contains an insertion of eight amino acids; the significance of this insertion is not known (10, 67). Finally, a key distinction between UBX mice and muscle TUG knockout (MTKO) mice, noted above, is that TUG cleavage products are generated only in UBX mice (12, 13). Alterations in glucose homeostasis were similar in UBX and MTKO mice, but energy expenditure was increased only in UBX mice. This phenotypic distinction led to studies to define a physiologic role of the TUG C-terminal product to regulate lipid oxidation and energy expenditure, as described below.

A few observations suggest that there may be a feed-forward circuit upstream of TUG cleavage, so that both positive and negative regulators of TC10α activation are stimulated by insulin. Such circuits can sense the rate-of-change of an input signal (68). In 3T3-L1 adipocytes, TC10α is activated between 2 and 5 min. after insulin addition, and is inactivated by 15 min. after insulin addition (62). This transient activation suggests that negative as well as positive regulators may be upstream, and that propagation of the negative signal is delayed relative to the positive signal. Individual proteins such as SH2B2 and c-Cbl may have multiple roles and contribute to this regulation (51, 69–72). Manipulation of specific protein functions and studies of tissue-specific knockout mice will be needed to understand signaling upstream of TC10α. Nonetheless, the data fit well with the idea that GLUT4 is released from a sequestered pool by a quantal mechanism, which is insulin dose-dependent (73, 74). The fraction of TUG that is cleaved is similarly insulin dose-dependent and can account for quantal release of discrete fractions of sequestered GLUT4 (11). Possibly, this is a physiologic mechanism to respond proportionately to the glycemic load of ingested nutrients.

Insulin signaling through Akt-independent mechanisms has received little attention. In part, this is because Akt signaling was suggested to be sufficient for GLUT4 translocation, based on studies using a chemical dimerizer to selectively activate Akt in 3T3-L1 adipocytes (75). These experiments showed that acute activation of Akt was able to increase plasma membrane localization of GLUT4. A caveat is that comparison was made to GLUT4 translocation after submaximal insulin stimulation (73). More importantly, it is uncertain whether GSVs were translocated, or whether the GLUT4 was recruited to the plasma membrane from endosomes, as it is during ongoing insulin exposure (16, 17). Movement of GLUT4 to the plasma membrane was delayed after acute Akt activation, compared to insulin stimulation; thus, the kinetics imply that GSVs were not well-mobilized by selective Akt activation (75). A similar delay in the recruitment of GLUT4 to the cell surface was observed using light-induced PI3K or Akt activation (76). It could be argued that, if Akt stimulates the dissociation of Tbc1D4 from IRAP, then this might contribute to the release of GSVs that are trapped intracellularly by Tbc1D4 together with TUG and other proteins (52). Yet, other data show that insulin acts in a PI3K-independent manner to stimulate the initial movement of GLUT4 to the cell periphery (33). Furthermore, in mice with muscle-specific deletion of Akt isoforms, effects on insulin stimulated glucose uptake were surprisingly minimal (77). In these studies, treatment with wortmannin, a PI3K inhibitor, ablated effects of insulin on *ex vivo* glucose uptake in both control and Akt knockout muscles. The results are consistent with a role for PI3K-dependent signaling through Rac1, but also suggest that other wortmannin-sensitive targets may be involved (4, 78).

Together, the data support the idea that both Akt-independent PI3K signaling and PI3K-independent insulin signaling are required for GLUT4 translocation (11, 13, 77). This conclusion applies in both fat and muscle cells. The data highlight the difficulty in interpreting early work using exogenous, overexpressed TC10α proteins in cell lines (79). Although studies in 3T3-L1 adipocytes and other cell lines can provide mechanistic insight, studies testing the requirement of specific pathways *in vivo* are needed to validate the physiological importance of specific points of regulation. Effects in MTKO mice support the centrality of the TUG cleavage pathway.

## Potential effects of exercise or muscle contraction

Additional signals may also act through TUG cleavage to cause GLUT4 translocation and may also modulate insulin sensitivity. Muscle contraction is well-known to stimulate GLUT4 translocation and glucose uptake, and also enhances subsequent insulin action (4, 80–82). These processes have been studied mostly in the context of the kinase AMPK, which phosphorylates the RabGAPs, Tbc1D4 and Tbc1D1, noted above. TUG is not known to be involved in this process, yet it is cleaved in cardiac muscle subjected to ischemia-reperfusion injury (83). Like muscle contraction, cardiac ischemia activates AMPK and causes GLUT4 translocation (84, 85). Sestrin2 is a scaffold protein interacts with the AMPK regulator LKB1, and deletion of Sestrin2 caused a reduction in ischemic AMPK activation, and reduced TUG cleavage and GLUT4 translocation. Thus, it seems likely that TUG cleavage may play a role in exercise- or contraction- induced GLUT4 translocation in skeletal muscle as well. Of note, GLUT4 and TUG abundances are highly correlated in muscles with diverse fiber types (86, 87). Finally, a potential AMPK-independent mechanism by which muscle contraction may be linked to TUG cleavage involves the TC10α -activating protein, obscurin, which interacts with several contractile proteins in the sarcomere (88, 89). If obscurin activates TC10α in a contraction-dependent manner, then this could be coupled through PIST and Usp25m to cause TUG cleavage and GLUT4 translocation.

Data suggest that the pool of GLUT4 that is translocated upon muscle contraction is distinct from that mobilized by insulin stimulation (4). Exercise- stimulated GLUT4 translocation is not impaired in insulin-resistant states; understanding this process may have therapeutic utility to increase GLUT4 translocation and glucose uptake in skeletal muscle. As well, how post-exercise insulin sensitivity is enhanced is not known. This effect may involve action through Tbc1D4, at least in part (82). Effects of TNKS to modulate the size of the pool of GSVs may also play a role, as discussed below (35, 36, 90). Further work will be required to understand whether TUG proteolysis mediates contraction-stimulated GLUT4 translocation in muscle, whether this acts on a distinct pool of GLUT4-containing vesicles, how this process affects exercise physiology, and how these processes are related to post-exercise insulin sensitivity.

## Ubiquitin-like proteolytic processing of TUG proteins

Insulin stimulates the ubiquitin-like processing of TUG proteins in 3T3-L1 adipocytes, as shown in **Figure 1** (11). Based on primary sequence alignments, intact TUG was noted to have a ubiquitin-like domain, now termed UBL1, at its N-terminus, as well as a UBX domain in its C-terminal region (7). The solution structure of UBL1 shows that it adopts a typical β-grasp fold (91). Early immunoblots of total membrane fractions from 3T3-L1 adipocytes, done using an antibody to the TUG C-terminus, detected not only the intact protein at 60 kDa, but also a 42 kDa band that had reciprocal intensity with the 60 kDa protein. This observation suggested a precursor-product relationship and prompted the hypothesis that TUG undergoes proteolytic cleavage (10). Specifically, it was hypothesized that TUG contains an additional ubiquitin-like domain, called UBL2 in **Figure 1**, and that it might be cleaved at the peptide bond linking residues Gly164 and Ser165. This might then produce a new ubiquitin-like protein modifier, termed TUGUL (for TUG Ubiquitin-Like). TUGUL contains a terminal diglycine sequence, which is typical of ubiquitin-like modifiers that can be covalently attached to target substrates (92, 93). By analogy to substrates that are ubiquitylated or sumoylated, the modified substrate would then be “tugulated” (10). Several ubiquitin-like modifiers are produced by maturation of larger precursor proteins; TUG is considered as a new precursor protein. The proteases that carry out this maturation process are deubiquitinating enzymes. These are identical to those that remove ubiquitin or ubiquitin-like proteins from their target substrates, and they cleave the peptide or isopeptide bond following the terminal glycine residue of the modifier.

Sequence alignments implied that TUGUL has two sequential ubiquitin-like domains, similar to other ubiquitin-like modifiers, ISG15 and FAT10 (10, 92, 93). Using antibodies to the TUG N- and C-termini, it was shown that endogenous TUG is cleaved and that TUGUL is incorporated into a single, 130 kDa protein in 3T3-L1 adipocytes. This protein was resistant to ionic detergent and its abundance and that of the C-terminal product were increased upon acute insulin stimulation. Because TUGUL is 18 kDa, it was hypothesized that a ~110 kDa target protein might be modified by attachment of a single TUGUL molecule. Subsequent data support the idea that this target is KIF5B, a kinesin motor that is upregulated during adipocyte differentiation and that carries GLUT4 to the cell surface (11, 33). Insulin acts by a PI3K-independent mechanism to stimulate both KIF5B-mediated GLUT4 movement and TUG cleavage (10, 11, 33). After insulin stimulation, TUGUL and KIF5B are present at 130 kDa and can be coimmunoprecipitated after denaturing lysis, and RNAi-mediated KIF5B depletion ablates the 130 kDa tugulated protein. Because the large intracellular loop of GLUT4 binds directly to TUGUL, likely within UBL2, it was considered that tugulation of KIF5B might load GLUT4 onto this motor (7, 11, 19). Supporting this idea, GLUT4 associated with KIF5B after insulin stimulation, and this association was abrogated by cleavage-deficient TUG mutants or by RNAi-mediated depletion of the TUG protease, Usp25m (see below). Of note, it has not been formally shown that this mechanism pertains in muscle, although the physiologic importance of KIF5B in adipose was shown in mice (94). As well, the site of TUGUL modification on KIF5B has not been identified, and it is not known if this modification affects motor activity. Even so, the data together support a model in which TUGUL modification of KIF5B links GLUT4 to these motors to promote the microtubule-based movement of GLUT4 to the cell surface.

**Figure 5:**
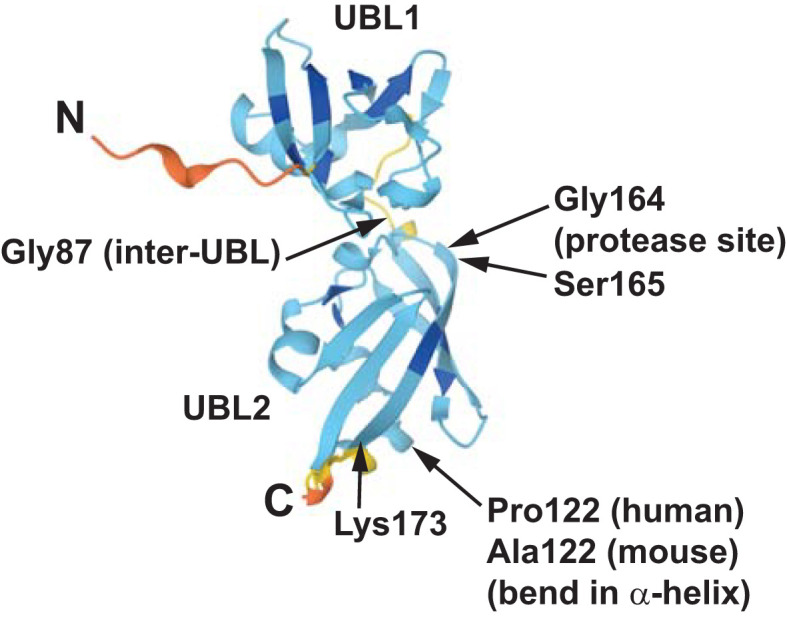
The structure of mouse TUGUL as predicted by AlphaFold is shown (accession Q8VBT9). The two ubiquitin-like domains, UBL1 and UBL2, are linked by a short peptide including the Gly87 residue. In both the mouse and human predicted structures, the main α-helix in UBL2 has a bend at residue 122, corresponding to Pro122 in humans and Ala122 in mice. The β-sheet that wraps around this helix has an extended central strand, extending to Lys173 in the predicted structure as shown. Data indicate that the scissile bond is that joining Gly164 and Ser165, which is near the beginning of this strand. This unusual structure may help to confer the cell type -specificity of TUG cleavage, which occurs primarily in fat and muscle cells. See text for details.

Recently, structure prediction using AlphaFold provided insight into the β-grasp topology of UBL2 (95, 96). The predicted structure of TUGUL is shown in **Figure 5** and demonstrates that there is a short peptide containing Gly87 that links the two ubiquitin-like domains. The predicted structure further identifies a bend in the major α-helix of UBL2, at residue Pro122 of human TUGUL or Ala122 of mouse TUGUL. The cleavage site, at the bond linking residues Gly164 and Ser165, is present at the beginning of the middle strand in the β-sheet that wraps around the helix. This is an unusual position for the scissile bond. A speculation is that when GLUT4 is bound, a peptide within the large intracellular loop of GLUT4 may substitute for this strand and make the scissile bond more accessible to the protease. This would be consistent with the observation that GLUT4 cotransfection enhances Usp25m-mediated TUG cleavage, and also enhances the ability of mature TUGUL to be used for covalent attachment in transfected cells (10, 11).

Data support the idea that Usp25m is the TUG protease. Usp25m is a splice variant of Usp25, which is expressed in muscle and adipose cells and contains an insertion of 70 residues compared to the more ubiquitous Usp25 splice form (also called Usp25a) (11, 97). The inserted residues facilitate the interaction of Usp25m with sarcomeric proteins and also contribute to its ability to cleave TUG in transfected cells (11, 98). Like IRAP, Usp25 proteins contain an RxxPDG motif that binds the ankyrin repeat domain of TNKS (42, 43). This interaction may position Usp25m on GSVs. In 3T3-L1 adipocytes, Usp25m is present on TUG-bound vesicles in unstimulated cells, and it dissociates from GLUT4 and TUG after insulin stimulation (11). Usp25m is also present together with intact TUG in light microsomes that have characteristics of lipid raft-like membranes, and the TUG C-terminal product is produced initially on these membranes. Other data show that Usp25m is required for TUG proteolysis, and insulin-stimulated GLUT4 translocation and glucose uptake (11, 42). Finally, cotransfection of Usp25m and sortilin causes TUG cleavage and GLUT4 translocation in transfected cells; coexpression of GLUT4 and Tbc1D4 enhanced the effect of Usp25m to cause TUG cleavage. It remains possible that Usp25m promotes a cycle of GLUT4 ubiquitylation and deubiquitylation, and that this is required for GLUT4 entry into GSVs (42). Yet, the simplest model to account for the data is that Usp25m acts directly to cleave TUG proteins and, thus, to mobilize GSVs to the cell surface.

Recent data show that Usp25 proteins form an auto-inhibited tetramer, and that disassembly of these tetramers activates the catalytic activity of these enzymes (99–102). Presumably, this model applies to Usp25m as well as to the ubiquitous Usp25 splice form. The unusual β-grasp fold present in the UBL2 domain of TUG may make it a substrate for Usp25m. Data imply that nPIST inhibits TUG proteolysis, as noted above, and that GTP-bound TC10α relieves this inhibition. One possibility is that nPIST stabilizes Usp25m tetramers in unstimulated cells, and that insulin acts through TC10α to reposition nPIST and to activate Usp25m activity. Usp25 regulation of TNKS stability has been studied because of its potential role in Wnt signaling (99, 103). Yet, in the context of GSV regulation, it may be more relevant to consider whether TNKS regulates Usp25m oligomerization. Possibly, TNKS could scaffold auto-inhibited Usp25m, so that these complexes can be activated by signaling through TC10α-nPIST.

The poly(ADP-ribose) polymerase activity of TNKS regulates insulin action in adipocytes, and we speculate that this results from an effect of poly(ADP-ribose) to promote biomolecular condensate formation (34, 36, 104). Such condensates could help to sequester GSVs intracellularly in unstimulated cells and, thus, to modulate insulin sensitivity. Of note, a similar role of liquid phase-separated condensates to cluster synaptic vesicles has been demonstrated (105–107). As well, Golgi matrix proteins, including Golgin-160 (which binds TUG), are described to form such condensates (108, 109). If this hypothesis is correct, then poly (ADP-ribose) -induced condensate formation could contribute to effects of NAD^+^ to enhance insulin action (110). Data from adipocytes and muscle cells, as well as from TNKS-deficient mice, are consistent with this model (35, 36, 90). As well, mass spectrometry shows that TUG is ADP-ribosylated on Ser538 (Ser535 in mice) (111). This modification may be reciprocally regulated with TUG acetylation, which also occurs near the C-terminus (32). Finally, initial studies in 3T3-L1 adipocytes observed variable modification of the TUG C-terminal cleavage product to a 54 kDa form, which remains uncharacterized (10). In subsequent work, the major C-terminal product was observed at the expected size of 42 kDa in primary muscle and in well-differentiated, mycoplasma-free 3T3-L1 adipocytes (11–13). Further studies in this area will be required to elucidate how an insulin signal is coupled to TUG proteolysis, the nature and role of modifications of the C-terminal product, and how this may be modulated by the metabolic state in fat and muscle cells.

By analogy to other ubiquitin-like modifiers, TUGUL must be acted upon by activating (E1), conjugating (E2), and possibly ligating (E3) enzymes to mediate its covalent attachment. The identities of these enzymes are unknown. In 3T3-L1 adipocytes, TUGUL modification occurs immediately upon TUG cleavage, and it seems most likely that the E1-E2-E3 enzymes are not direct targets of insulin signaling. TUGUL-modified KIF5B was observed in cells treated with wortmannin, indicating that if an additional signal is required, this is not PI3K-dependent (11). GLUT4 and IRAP may serve as components of a TUGUL E3, in that they bind TUGUL and may bring it into proximity of other enzymes. In particular, the SUMO conjugating enzyme, Ubc9 (UBE2I) was shown to bind to the C-terminal tail of GLUT4 and to control its trafficking and, likely secondarily, its stability (112, 113). Although GLUT4 may be sumoylated, another possibility is that Ubc9 functions in this context as a TUGUL E2 (10, 114). The TUGUL C-terminus most closely resembles that of SUMO, among other ubiquitin-like protein modifiers, and it may therefore interact with Ubc9. Of note, Ubc9 hemizygous knockout mice had a slight but significant increase in glycemia (115). The abundances of GLUT4 and Ubc9 proteins were reduced in muscle of individuals with severe insulin resistance (116). Thus, data suggest that Ubc9 may function as a TUGUL E2.

## The p97 (VCP) ATPase, membrane trafficking, and cell type -specificity

The TUG C-terminal region contains a UBX domain. Like other proteins containing this domain, TUG binds to the hexameric ATPase p97, also called VCP (117–119). Yet, TUG lacks a key phenylalanine residue that mediates p97 binding by UBX domains in other proteins, and instead contains a proline at this position (residue 433 in mice) (120, 121). Accordingly, the TUG UBX domain does not bind avidly to p97 (117). Binding is mediated by an extended UBX (eUBX) region containing structural elements both N- and C-terminal to the UBX domain itself (117, 119, 122–124). Within the TUG UBX domain, Pro433 (Pro437 in humans) forms a *cis*-Pro touch-turn motif that interacts with the p97 N-terminal domain to facilitate disassembly of p97 hexamers. This disrupts p97 ATPase activity *in vitro* and suggests that TUG may regulate p97 ATPases by controlling their oligomerization state within cells. Other data show that TUG recruits a lysine methyltransferase to p97, and imply that p97 methylation regulates its ATPase activity. Specifically, METTL21D (also called VCP-KMT) binds to both TUG and p97, and TUG stimulates the methylation of p97 by METTL21D (125, 126). The modified residue, Lys315, is trimethylated and this modification reduces p97 ATPase activity *in vitro*. Although METTL21D-mediated methylation appears to be highly specific for p97 Lys315, the importance of this as a site of regulation *in vivo* remains uncertain (127). METTL21D knockout mice had no obvious pathological phenotype and apparently normal running capacity, compared to wildtype controls (127). Of note, recent proteomic data identified p97 as a major interacting partner of Tbc1D4 in human and mouse skeletal muscle (128). Thus, although TUG may regulate p97 activity *in vivo*, precisely how this occurs and whether it is important for GSV regulation remains uncertain.

The p97 ATPase is well-known to extract individual proteins from protein complexes, and it plays important roles as an unfoldase and in membrane fusion (129). TUG may participate in one or more of these processes, either by regulating p97 ATPase activity and/or by serving as an adaptor or substrate for p97. In adipocytes, after insulin-stimulated TUG cleavage, p97 activity is required to extract the C-terminal cleavage product and to target it to the nucleus (13). In HeLa cells, intact TUG localizes to the endoplasmic reticulum -Golgi intermediate compartment (ERGIC), endoplasmic reticulum exit sites (ERES), and the cis-Golgi compartment (117). In these studies, it was estimated that 5-10% of cellular p97 was bound to TUG, and that 85-90% of TUG was bound to p97. Thus, one possibility is that TUG regulates p97 function at the early secretory pathway. Consistent with this, reformation of the Golgi after Brefeldin A removal was delayed in cells with siRNA-mediated TUG depletion. The specific mechanism by which TUG and p97 act at the early secretory pathway remains unknown, but p97 is coupled to membrane fusion processes though adaptors such as p37 (UBXN2B), which acts with p115-GM130 to promote Golgi membrane fusion (130). This may be relevant to GSV regulation, because p115 interacts with IRAP to control GLUT4 trafficking (131). It seems possible that hexameric p97 ATPase may interact with more than one adaptor at the same time, and so the idea that TUG and p37 (or another adaptor) might function together is not unreasonable. Understanding how a general trafficking pathway (e.g. for Golgi assembly) may be adapted in a cell type -specific manner (e.g. for insulin-stimulated TUG cleavage and GLUT4 translocation) will require further studies.

The localization of TUG at the ERGIC, ERES, and cis-Golgi led us to propose that insulin stimulates GSV mobilization along an unconventional secretion pathway, which bypasses the Golgi complex (1, 27, 132). This idea draws on data showing that GSVs fuse directly with the plasma membrane (16, 17). Other recent data suggest that insulin triggers the fusion of GLUT4-containing vesicles with endosomes; whether these vesicles are GSVs and, if so, what fraction of GSVs behave this way, remains uncertain (133). The idea that TUG acts at the ERGIC is further supported by data showing that it functions with Golgin-160 and ACBD3, which localize to the early secretory pathway (32). In human cells, insulin-responsive GLUT4 trafficking requires a specific clathrin, CHC22, which is also localized to the early secretory pathway (45). Of note, TC10α and PIST regulate the trafficking of CFTR, particularly the ΔF508 mutant, which is also proposed to reach the plasma membrane by a similar, unconventional secretion pathway (134–138). Characterization of this pathway may reveal how it regulates various membrane proteins and will likely have broad relevance for physiology and disease.

## Coordinated regulation of distinct physiologic effects

Studies in mice revealed that TUG proteolytic processing coordinates the regulation of various physiologic processes. As noted above, data support the idea that TUG regulates the main step in GLUT4 translocation, and that its action accounts for a large fraction of the overall ability of insulin to control glucose uptake in muscle (12, 13). In addition to GLUT4, TUG-regulated GSVs contain IRAP, which regulates vasopressin action, and LRP1 and sortilin, which bind ApoE and ApoA5 lipoproteins and may participate in lipid metabolism (2, 22, 27). Effects due to the translocation of these proteins are coordinated with actions of the TUG C-terminal cleavage product, which regulates lipid oxidation and energy expenditure (13). **Table 1** summarizes these effects and **Figure 4** depicts how these distinct actions are coordinately regulated by TUG endoproteolytic cleavage.

Initial data to elucidate effects on water homeostasis were obtained using UBX mice, which have constitutive, insulin-independent TUG cleavage in muscle (22). Unexpectedly, these mice had markedly increased water intake, compared to controls. Water intake was increased by 55%, arguably a greater effect than that on whole body fasting glucose turnover, which was increased by 17% in the transgenic animals (12, 22). In MTKO mice, water intake was increased by 29%, which is comparable to the 27% increase in fasting glucose turnover that was observed. IRAP acts physiologically to cleave and inactivate vasopressin (139). Therefore, it was hypothesized that the translocation of IRAP to T-tubule membranes in UBX mice caused accelerated vasopressin inactivation. If increased vasopressin secretion was unable to fully compensate, then the mice would have a partial diabetes insipidus (inability to concentrate urine). Supporting this model, UBX mice had evidence of both decreased vasopressin action and increased vasopressin secretion. Increased water intake was also observed in MTKO mice, and increased targeting of IRAP to T-tubules was observed in both models (13, 22). Of note, the half-life of circulating vasopressin is likely ~1 min. in both humans and mice (2). This half-life is prolonged ~3-fold in IRAP knockout mice and shortened ~3-fold in UBX mice (22, 139). From a physiologic standpoint, vasopressin inactivation may increase muscle perfusion, so that the delivery of glucose is coordinated with glucose uptake. This mechanism is independent of nitric oxide. Finally, in insulin resistant individuals, altered targeting of IRAP may contribute to hypertension, as described (2).

A further unexpected observation was that UBX mice had increased energy expenditure, compared to controls (12). This could not be attributed to increased glucose uptake itself. Rather, the effect might result from translocation of another GSV cargo (given the precedent of IRAP, above), or from effects of the TUG C-terminal cleavage product. MTKO mice were used to distinguish these possibilities, as TUG deletion was predicted to cause translocation of GSV cargos without the generation of cleavage products (13). In contrast to UBX mice, MTKO mice had unchanged (on regular chow) or reduced (on a high fat diet) energy expenditure, supporting the idea that the C-terminal cleavage product mediates this effect. In agreement with data from transfected cells, the endogenous product enters the nucleus (13, 117). Transcriptome analyses of UBX muscle revealed upregulation of genes that mediate fatty acid oxidation and thermogenesis, and implied that this program is regulated by the PPARγ transcription factor. Indeed, the TUG C-terminal product binds PPARγ directly, and recruits a co-factor, PGC-1α, which controls the expression of genes regulating mitochondrial function. Two specific proteins that are regulated by this pathway are sarcolipin (Sln) and uncoupling protein 1 (Ucp1), which function mainly in muscle and adipose, respectively. Sln uncouples ATP hydrolysis from calcium transport into the sarcoplasmic reticulum, whereas Ucp1 uncouples the mitochondrial inner membrane protein gradient from ATP synthesis; in both cases, thermogenesis is increased (140, 141). In addition, transcriptome data from UBX quadriceps reveal increases in *Slc25A1*, *Acly*, *Acaca*, and *Fasn*, suggesting that flux through the citrate-malate shuttle is increased (13, 142). This is predicted to result in generation of cytosolic acetyl-CoA and enhanced lipogenesis. This lipogenic effect could result either from action of a GSV cargo or from the TUG product. Together, the data support the hypothesis that action of the TUG C-terminal product contributes to the thermic effect of food, a transient increase in energy expenditure that occurs after meals (143).

Like that of other C-terminal cleavage products, the stability of the TUG product is regulated by an N-degron pathway (144). In mice, induced whole-body deletion of ATE1, which regulates one such pathway, results in increased energy expenditure and loss of fat mass (145). ATE1 is an arginyl-tRNA-protein transferase that attaches an Arg residue to the N-terminus of proteins that begin with Asp, Glu, or oxidized Cys residues. Arginylation creates a signal that is recognized by proteosomal or autophagic machinery, leading to degradation of the arginylated product. The TUG C-terminal product begins with a Ser residue (Ser165 of intact TUG), and so would not normally be considered as a physiologic substrate for the Arg/N-degron pathway. Yet, the product was stabilized in ATE1 knockout cells, showing that it is regulated by this pathway (13). Of note in wildtype cells, the Ser-containing TUG product was not fully destabilized and had a half-life of ~2 h; this was increased to ≥16 h in ATE1 knockout cells. Finally, PGC-1α also has a relatively short half-life (~12 min) and was stabilized by the TUG product; in ATE1 knockout cells cotransfected with both proteins, the half-life of PGC-1α was also prolonged. The data support the idea that the duration of the thermic effect of food may be regulated by an ATE1-dependent degradation pathway. It is not known whether ATE1 acts directly on the TUG product, and the effect of ATE1 knockout could be indirect. However, another possibility might be that the N-terminal Ser residue could be subject to oxidation by highly reactive oxygen species (146). If so, this could make it susceptible to arginylation, by analogy to oxidized Cys (147). For this to occur, the Ser would likely have to reside in a context in which it could be deprotonated, so that a peroxide could be formed; structural studies may be revealing. Alternatively, mitochondrial reactive oxygen species might somehow signal through phosphorylation to control the stability of the TUG product (148). Regardless, it seems likely that the stability of the TUG C-terminal product could be a point of regulation to control oxidative metabolism, and further studies in this area may be informative.

## Implications for metabolic disease

Human genetic variations may affect diabetes risk by modulating the stability of the TUG C-terminal product. A polymorphism in the coding region of PPARγ2 results in proteins containing Ala or Pro residues at position 12, and individuals with Ala12 are protected from type 2 diabetes (149, 150). Because of the high prevalence of this polymorphism, it is estimated that there would be 18-20% less diabetes in the population if only the Ala12 allele were present. The TUG C-terminal product binds directly to the PPARγ2 N-terminal peptide, and interacts more avidly with the Ala12 variant, compared to the Pro12 variant (13). It is hypothesized that this interaction helps to recruit the PGC-1α coactivator and that it stabilizes the complex. Individuals with the PPARγ2 Ala12 variant would then have an increased thermic effect of food and might be less likely to develop diabetes on that basis. The thermic effect of food is difficult to measure, and small variations may have cumulative effects over extended periods of time. Therefore, testing this hypothesis definitively may be technically challenging. Genetic variations in *CAPN10*, encoding the calpain-10 protease, also modulate diabetes risk, and data imply that calpain-10 may also act on intact TUG or on the TUG C-terminal product (151). Physiologic studies will be required to test if this affects metabolism. Variation in ankyrin-b (AnkB) also modulates diabetes risk and may affect GLUT4 translocation through effects on TUG and obscurin, as noted above (88, 89, 152). In skeletal muscle, the magnitude of GLUT4 translocation and glucose uptake are greater after contraction, compared to insulin stimulation (153). If TUG cleavage is involved in this process, then effects of the C-terminal cleavage product may contribute to excess post-exercise oxygen consumption (154). Genetic variations that modulate this pathway would then impact energy expenditure after exercise, as well as after meals. Inasmuch as genetic polymorphisms implicate pathways that are important for pathophysiology, and not just individual genes or interactions, these genetic variations highlight the importance of understanding the TUG cleavage pathway as it relates to common metabolic disease.

It is hypothesized that for TUG to be susceptible to cleavage, the GSVs must exist as a distinct population of vesicles that are linked to the Golgi matrix by intact TUG. In insulin-resistant individuals, the targeting of GLUT4 and IRAP is altered during fasting (155, 156). This effect is independent of an insulin signal, and therefore may account for insulin resistance that cannot be attributed to attenuated signaling (157, 158). One possibility is that the GSV cargoes remain trapped in Golgi or other membranes of the early secretory pathway. This could result in destabilization of Usp25m and impaired TUG cleavage, as observed in muscle and adipose tissues of rodents with diet-induced insulin resistance (11, 13). Of note, ceramides implicated in insulin resistance alter GLUT4 sorting through a syntaxin-6 compartment in muscle (159). Since syntaxin-6 is likely present in TUG-bound GSVs, it is easy to imagine that this altered sorting may reduce GSV formation, TUG cleavage, and Usp25m abundance (10). Other data implicate specific ceramides in selective sorting of cargoes at the early secretory pathway (160). Such effects may be independent of diacylglycerol-dependent mechanisms that impair insulin signaling (161). The idea that insulin resistance may involve a distal step, closely linked to the terminal biological response, glucose uptake, is consistent with recent analyses (157, 162). From a practical standpoint, studies of insulin resistance would be well served by immunoblotting for TUG cleavage products, possibly in addition to phosphorylated Akt or Tbc1D4 proteins. As noted above, the number of TUG proteins that are cleaved is proportional (and possibly equal) to the number of GSVs that are translocated; the GSVs themselves may each contain a narrowly defined number of GLUT4 proteins (10, 21). Thus, TUG cleavage and GLUT4 translocation may be related by stoichiometry. Finally, a better understanding of GSVs, their subcellular location and architecture, and of how these vesicles are affected in insulin-resistant states, will likely be informative for pathophysiology.

A truncated form of TBC1D4 confers insulin resistance and increases diabetes and cardiovascular risk in the Inuit population in Greenland (163, 164). This effect has been attributed exclusively to effects on GLUT4 and glucose uptake. Yet, if the Tbc1D4 protein acts together with TUG to regulate GSVs, as described above, then there may also be effects due to altered targeting of IRAP or other GSV cargoes. It would be interesting to know whether the altered trafficking of these cargoes results in reduced TUG cleavage, with consequently reduced energy expenditure. Rodent data suggest that insulin-resistance may cause reduced post-prandial energy expenditure, resulting from decreased production of the TUG C-terminal cleavage product (13). In humans, the thermic effect of food is reduced in obesity, which is consistent with this idea (165, 166). If insulin resistance causes reduced energy expenditure in humans, then a cycle may result in which obesity causes insulin resistance, which promotes further obesity and worsened insulin resistance. Improved understanding of these processes may thus have broad ramifications for prevention and treatment of metabolic disease.

## Author contributions

The author confirms being the sole contributor of this work and has approved it for publication.

## Funding

Research in the author’s laboratory is supported by NIH R01 DK129466 and by the Yale Diabetes Research Center, NIH P30 DK045735.

## Acknowledgments

The author thanks Drs. Don Li, Estifanos Habtemichael, Derek Toomre, Richard Kibbey, Rachel Perry, and Gerald Shulman for helpful discussions.

## Conflict of interest

The author declares that the research was conducted in the absence of any commercial or financial relationships that could be construed as a potential conflict of interest.

## Publisher’s note

All claims expressed in this article are solely those of the authors and do not necessarily represent those of their affiliated organizations, or those of the publisher, the editors and the reviewers. Any product that may be evaluated in this article, or claim that may be made by its manufacturer, is not guaranteed or endorsed by the publisher.
